# Unraveling the Effect of Singlet Oxygen on Metal-O_2_ Batteries: Strategies Toward Deactivation

**DOI:** 10.3389/fchem.2020.00605

**Published:** 2020-07-22

**Authors:** Idoia Ruiz de Larramendi, Nagore Ortiz-Vitoriano

**Affiliations:** ^1^Inorganic Chemistry Department, University of the Basque Country UPV/EHU, Bilbao, Spain; ^2^Center for Cooperative Research on Alternative Energies (CIC EnergiGUNE), Basque Research and Technology Alliance (BRTA), Vitoria-Gasteiz, Spain; ^3^Ikerbasque, Basque Foundation for Science, Bilbao, Spain

**Keywords:** metal-O_2_ batteries, singlet oxygen, quencher, superoxide, parasitic side reactions

## Abstract

Aprotic metal-O_2_ batteries have attracted the interest of the research community due to their high theoretical energy density that target them as potential energy storage systems for automotive applications. At present, these devices show various practical problems, which hinder the attainment of the high theoretical energy densities. Among the main limitations, we can highlight the irreversible parasitic reactions that lead to premature death of the battery. The degradation processes, mainly related to the electrolyte, lead to the formation of secondary products that accumulate throughout the cycling in the air electrode. This accumulation of predominantly insulating products results in the blocking of active sites, promoting less efficiency in system performance. Recently, it has been discovered that the superoxide intermediate radical anion is involved in the generation of the reactive oxygen singlet species (^1^O_2_) in metal-O_2_ batteries. The presence of singlet oxygen is intimately linked with electrolyte degradation processes and with carbon-electrode corrosion reactions. This review analyzes the nature of singlet oxygen, while clarifying its toxic role in metal-O_2_ batteries. Besides, the main mechanisms of deactivation of singlet oxygen are presented, trying to inspire the research community in the development of new molecules capable of mitigating the harmful effects related to this highly reactive species.

## Introduction

In a world increasingly concerned with the dire consequences of climate change, the increase in renewable energy generation over the last 20 years offers hope in the struggle to save the planet. Thus, satisfying an ever-growing thirst for energy—which doubled between 1971 and 2016—via sustainable technologies from renewable sources represents one of the most significant global challenges of this century. One of the most difficult hindrances is related to the intermittent nature of the main renewable sources—such as wind or sun—that is a driving force behind the need for new storage technologies. Among these different storage systems, those based on electrochemical reactions offer the greatest potential to have an impact on generation, storage and consumption at a global level. Lithium-ion batteries (LiBs), first developed in 1991, paved the way for the changes, which followed, and revolutionized the market of portable electronic devices thanks to their high volumetric and gravimetric energy density. However, the continuous growth of the LiB market has led to a large increase in the demand and price of lithium—a metal of limited availability and restricted geographical distribution. As a result, a growing interest exists in the development of alternative, “beyond lithium” technologies which may improve upon and even replace LiBs. Within this scenario, Metal-air/O_2_ batteries (MaBs) where cell chemistries include Li-O_2_, Na-O_2_ and K-O_2_, have the potential to diversify and revolutionize both stationary and mobile applications due to their low cost and scalability (when compared to Li-ion technology). In this mini-review Li and Na variants will be discussed due to their inherent excellent properties. These MaBs present theoretical energy densities much higher (based on discharge products Li_2_O_2_, 3,505 W h kg^−1^; Na_2_O_2_/NaO_2_: 1,605/1,108 W h kg^−1^, respectively) than that of Li-ion batteries (100–300 Wh kg^−1^) (Landa-Medrano et al., 2016a; Enterría et al., 2018). Moreover, unlike traditional intercalation-based battery systems, MaBs rely on the electrochemical reduction of molecular oxygen at the cathode surface which translates into negligible amount of “dead weight.” In addition, carbon-based cathode materials are commonly used which are cheap and environmentally friendly (Munuera et al., 2020).

MaB mechanism relies on the formation and decomposition of the discharge products during the charge/discharge processes, according to the following equations:

Anode (discharge):    *M* ↔ *M*^+^+ *e*^−^

Cathode (discharge):    $xM^{+}+\;O_{2}+xe^{-}\;↔\;M_{x}O_{2}$

                                    where x=1 or 2

Although MaBs present the above mentioned properties, their application today is hampered by the sluggish kinetics of oxygen reduction (ORR) and oxygen evolution (OER) reactions (Landa-Medrano et al., 2016a) and the parasitic reactions occurring during cycling. One of the main challenges is related to their poor charge reversibility due to the formation of parasitic reactions (superoxide intermediates and radicals) and secondary phases (e.g., carbonates, hydroxides) which degrade the battery components during cycling (Lai et al., 2020; Yadegari and Sun, 2020) and are electrochemically irreversible (Landa-Medrano et al., 2016b, 2017, 2019). These reactions can lead to the formation of alkali acetates, carbonates and carboxylates, among others, which are hard to oxidize; thus, these species accumulate on the cathode surface leading to the premature cell death (Aurbach et al., 2016). Although initially the formation of these by-products seemed to be linked to the presence of the superoxide species that promoted the breakdown of the electrolyte, it has recently been shown that such decomposition is accelerated by the existence of the singlet molecular oxygen species (^1^O_2_) as a reaction intermediate (Mahne et al., 2017).

In this mini-review the main generation routes of singlet oxygen in MaBs will be summarized, together with the consequences for the battery performance. The proposed mechanisms for its generation and the effect of its presence on the electrolyte on the performance of the battery will also be analyzed critically. A range of approaches will be proposed in order to inhibit the negative effects related to the presence of singlet oxygen. Finally, remaining challenges and prospects will be discussed, providing a perspective on future trends.

## What is Singlet Oxygen?

**Figure 1:**
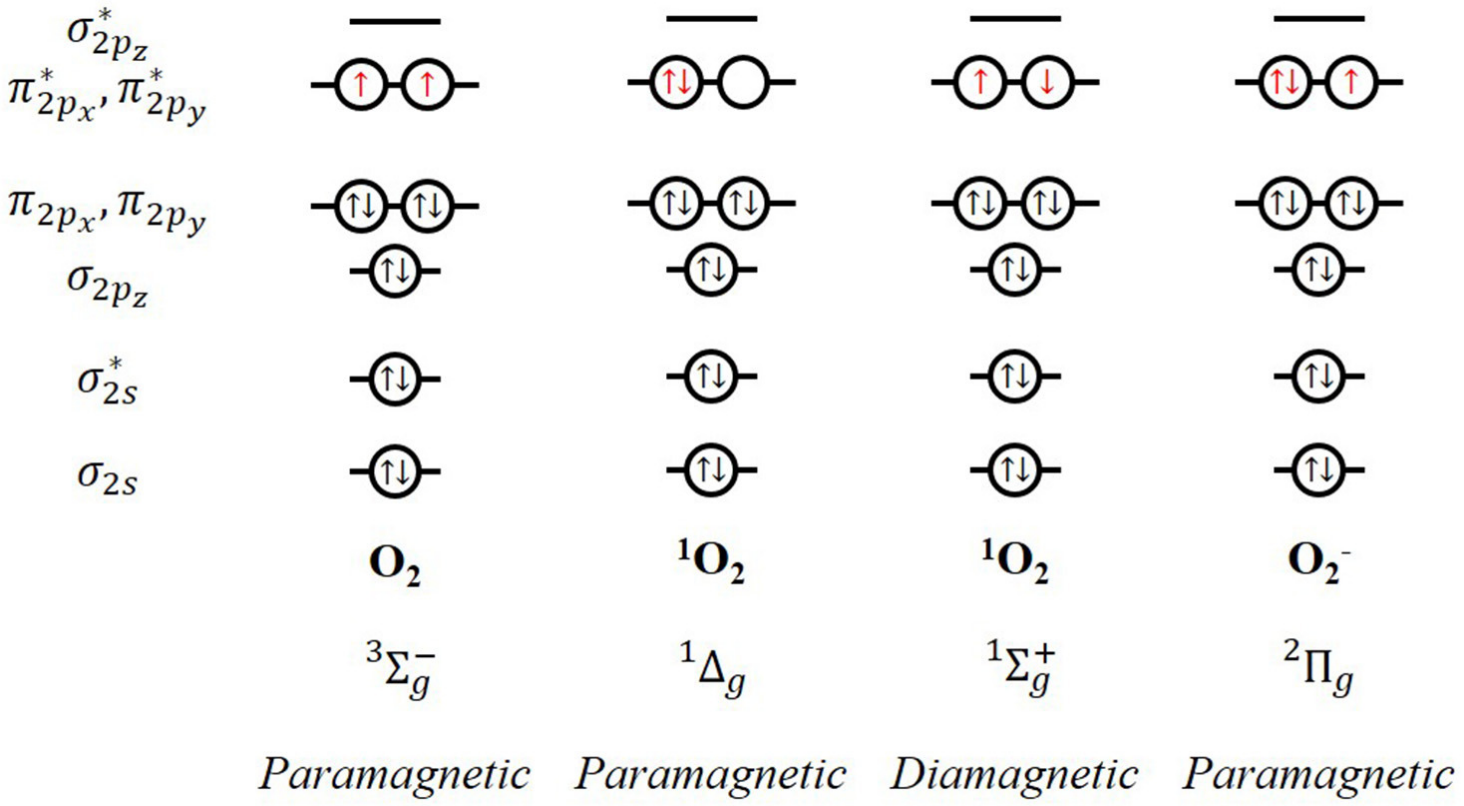
Molecular orbitals and magnetic response of triplet and singlet dioxygen and superoxide radical anion.

Oxygen is ubiquitous. The oxygen molecule in its ground state presents an even number of electrons in anti-bonding degenerate π orbitals, which translates into a series of quite unusual properties regarding its magnetic and spectroscopic response and its reactivity, among others. This is ascribed to the open-shell electronic structure of the molecule, where two oxygen atoms with six valence electrons each get bounded. Applying the molecular orbital theory, the electronic configuration of the oxygen molecule is $KK{\left(\sigma_{2s}\right)}^{2}{\left(\sigma_{2s}^{*}\right)}^{2}{\left(\sigma_{2p_{z}}\right)}^{2}{\left(\pi_{2p_{x}},\pi_{2p_{y}}\right)}^{4}{\left(\pi_{2p_{x}}^{*},\pi_{2p_{y}}^{*}\right)}^{2}$. According to Mulliken, this electronic configuration is associated with three electronic states which are energetically very close (Mulliken, 1928, 1929). The energy of the triplet state (Σ 3g−) is the lowest with two unpaired electrons in the highest occupied molecular orbitals. Triplet ground state molecular oxygen is a paramagnetic biradical with two electrons occupying separate π^*^ orbitals with parallel spins (Figure 1). Rearrangement of the electron spins within these two degenerate π ^*^ orbitals results in two possible singlet excited states (Kearns, 1971):

- Δg 1: both electrons are paired in a single π ^*^ orbital, leaving the other vacant. This spin-paired electronic state was first observed in 1924 by Lewis. The spin restriction is removed so that the oxidizing ability is significantly increased in comparison to the ground state. This first excited state presents an energy of 22.5 Kcal above that of the triplet ground state dioxygen (Σ 3g−), with a lifetime of 45 min under ideal conditions, which is reduced to about 10^−3^ s in a real solution environment. This is enough to play an important role in chemical reactions in solution; it is expected to participate in two-electron reactions. In fact, this state is known as metastable O_2_ species, which is commonly called singlet oxygen (^1^O_2_).
- Σ 1g+: there is a pairing of the spins of the two electrons which are located in different π^*^ orbitals. This configuration might result in one-electron free-radical reactions. This state has a greater energy above aground state (38 Kcal) and considerable shorter lifetime (10^−9^ in real conditions). In fact, decays to Δg 1 before a chemical reaction is initiated.

Both physical and chemical methods can generate singlet oxygen. Physical methods include photosensitization by activation of the light in the visible and UV regions; and, microwave or radiofrequency discharge in an oxygen atmosphere, among others. On the other hand, the main chemical sources of singlet oxygen are related to the deactivation of different compounds such as hydrogen peroxide, ozonides, or endoperoxides. Thus, the superoxide ion (a key intermediate in most metal-O_2_ batteries) may be involved in the production of ^1^O_2_. The electronic configuration of superoxide radical anion is provided in Figure 1. The loss of an electron with the proper spin can give rise to both the ground state of molecular oxygen and the excited state of singlet oxygen, as presented in Equation 1:

(1) O 22−→O 1,32+e−

It is, therefore, of utmost importance to analyze and estimate the role of ^1^O_2_ in the development of future MaBs, due to the implication of superoxide in the generation of singlet oxygen and, above all, the great oxidizing capacity of this reactive species.

## What are the Implications for Metal-O_2_ Batteries?

**Figure 2:**
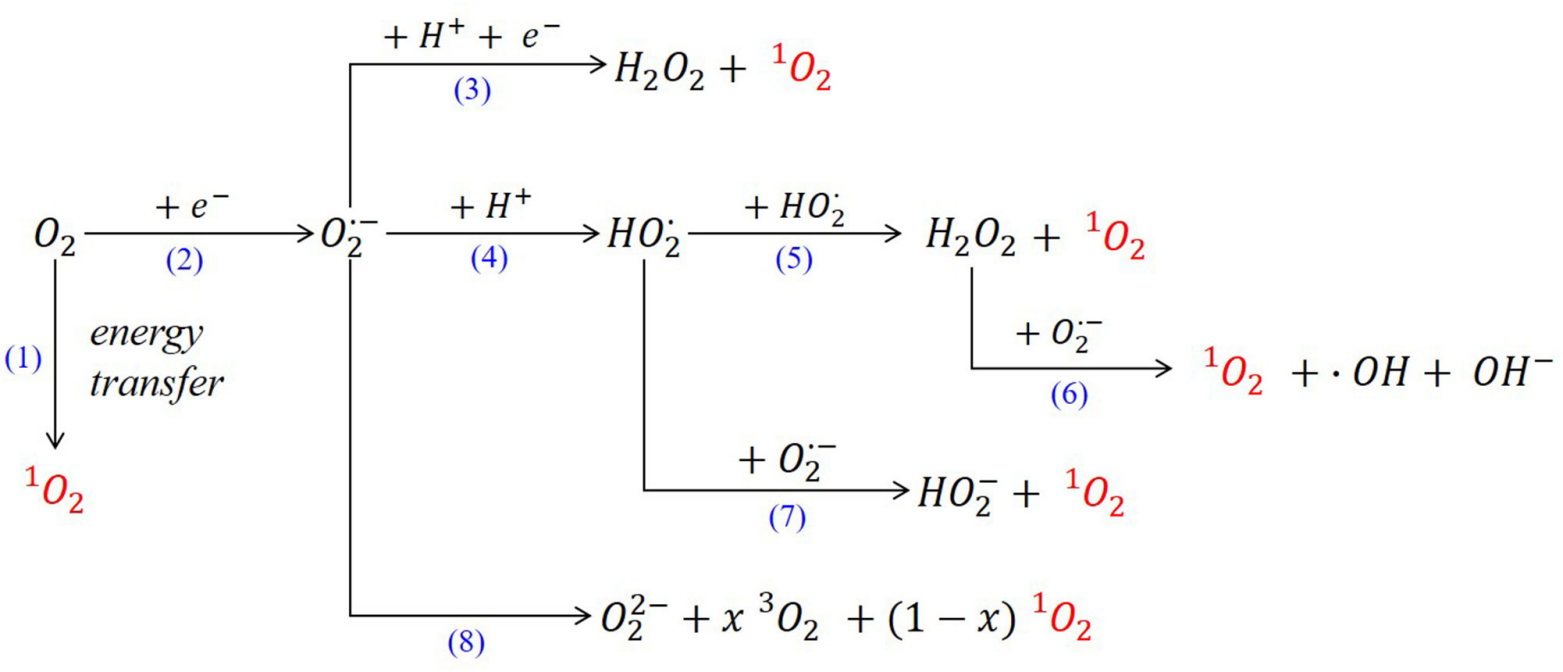
Main mechanisms of singlet oxygen formation in metal-O_2_ batteries.

Initially, the formation of the parasitic products was associated to the presence of superoxide, which promotes electrolyte decomposition; however, recently it has been demonstrated that decomposition and parasitic side reactions are accelerated by the presence of singlet oxygen as reaction intermediate (Hassoun et al., 2011; Mahne et al., 2017). Actually, ^1^O_2_ is also related to the main cause of the deactivation of organic redox mediators, leading to a decrease in the catalytic effect during Li-O_2_ battery cycling (Kwak et al., 2019). The presence of this strong oxidizing agent was first detected in Li-O_2_ batteries by in operando electron paramagnetic resonance (EPR) using 2,2,6,6-tetramethyl-4-piperidone (4-Oxo-TEMP) as a spin trapping agent to form a stable radical, 2,2,6,6-tetramethyl-4-oxo-piperidin-1-oxyl (4-oxo-TEMPO) (Wandt et al., 2016). Later, its formation was monitored by fluorescence measurements using 9,10-dimethylanthracene (DMA) quencher (Mahne et al., 2017). In both studies, the degradation of the electrolyte and the cathode was ascribed to the presence of parasitic side reactions related to the presence of ^1^O_2_. During charge process in Li-O_2_ batteries, the oxygen released was involved in the degradation of the cell, due to the corrosion of the carbon at potentials >3.5 V (vs. Li^+^/Li). Wandt et al. demonstrated that this degradation was ascribed to the presence of intermediate species such as the generation of singlet oxygen at charge potentials between 3.55 and 3.75 V (vs. Li^+^/Li) (Wandt et al., 2016). The latest studies on the mechanisms involving the generation of singlet oxygen in Li-O_2_ batteries, conclude that the major source of ^1^O_2_ is the disproportionation (also known as dismutation) of LiO_2_ (Figure 2, reaction 8) through the following mechanism (Córdoba et al., 2019; Mourad et al., 2019; Wang and Lu, 2020):

(2 2LiO2→(LiO2)2→Li2O2+O 12

Regarding Na-O_2_ batteries, the superoxide cannot be solely responsible for the formation of side reactions; the formation of ^1^O_2_ has been also observed using DMA quencher, being present in higher quantities at high potentials (Schafzahl et al., 2017). The main mechanisms of ^1^O_2_ formation in metal-O_2_ batteries are summarized in Figure 2.

In presence of aprotic solvents, the one-electron reduction of oxygen leads to the formation of the superoxide radical anion (Figure 2, reaction 2). Once this anion is formed through the dismutation reaction, hydrogen peroxide and singlet oxygen are form in presence of protons (Figure 2, reaction 3) (Fridovich, 1972). Stauff et al. analyzed further the emission of the singlet oxygen formed in the superoxide dismutation reaction in the presence of proton donors; this new route involves the formation of the perhydroxyl radical intermediate (Figure 2, reactions 4–7) (Stauff et al., 1963). Often the proposed mechanisms in the literature show that the formation of ^1^O_2_ is linked to the presence of acidic protons whose source in numerous studies is water (Andrieux et al., 1987; Mahne et al., 2017; Schafzahl et al., 2017). Actually, the effect of the presence of water on the reaction mechanism has already been analyzed in metal-O_2_ batteries by some researchers. Nazar et al. proposed a proton phase-transfer catalysis (PPTC) which is critical to solubilizing and transporting the superoxide (Xia et al., 2015). This study showed that the controlled presence of water in the electrolyte (up to 100 ppm) led to an increase in the battery capacity where larger NaO_2_ cubes were formed. This effect was attributed to the presence of H^+^ in H_2_O that participate in the crystal growth, not as solvation of the superoxide but as *proton phase-transfer catalysis* (PPTC). The formation of $HO_{2}^{\cdot }$ intermediate (Figure 2, reaction 4) might reduce the formation of parasitic reactions, leading to a higher discharge capacity (Qiao et al., 2017). In Na-O_2_ batteries, this PPTC mechanism implies that the proton would act as a carrier, transporting the superoxide from the cathode surface to the electrolyte, where the superoxide would interact with the sodium cations releasing the proton again via metathesis reaction (Equation 3):

(3) $$\begin{array}{r} HO_{2}\;+Na^{+}⇌NaO_{2}+H^{+} \end{array}$$

Similarly, it is well-known that H_2_O can promote a solution-mediated pathway (Kwabi et al., 2016) but could also reduce the reversibility in aprotic Li-O_2_ batteries (Ma et al., 2018). Thus, it has been established that the presence of very low water concentrations (below 100 ppm) can improve the performance of Na-O_2_ systems. However, when the water content is higher, a faster disproportionation reaction is facilitated, leading to the formation of singlet oxygen. In fact, the presence of proton donor species, such as ammonium Brönsted acid, has recently been determined to favor increased ^1^O_2_ generation (Lozano et al., 2020).

Lately, formation of singlet oxygen by the disproportionation of superoxide anion in the presence of other cations in the electrolyte with different Lewis acidity has been evaluated (Mourad et al., 2019). Cations with weak Lewis acidity favor the formation of ^1^O_2_, which is especially important when developing new electrolytes based on ionic liquid cations such as imidazolium. In the same way, the disproportionation reactions of LiO_2_ and NaO_2_ discharge products can also act as a source of singlet oxygen. Based on this study, the main problem related to ^1^O_2_ arises; the disproportionation reaction is necessary to obtain high capacity in these systems, which allows the decomposition of the discharge products, but also acts as a source of singlet oxygen, which, in turn, leads to irreversible reactions. If the singlet oxygen is responsible for the cell irreversibility, it is necessary to develop new strategies able to deactivate this species while allowing disproportionation, without altering the (electro)chemistry of the battery.

## How Singlet Oxygen Can be Deactivated?

Deactivation of the excited state of the dioxygen molecule can be accomplished by either chemical or physical quenching. In the first case, singlet oxygen reacts with quencher R to produce RO_2_, which implies oxygen consumption. An example of a chemical quencher is DMA which after reaction with ^1^O_2_ forms the corresponding endoperoxide. Regarding its application in metal-O_2_ batteries, however, its use is not convenient, since the consumption of O_2_ within the battery might lead to its premature death. In addition, the formation of intermediate species of an insulating nature will cause a blockage of active sites in the air electrode, leading to a decrease in capacity with cycling. In the physical process, by contrast, there is neither consumption of oxygen nor formation of secondary products which maintains the quencher without chemical changes. Two major physical mechanisms are known, namely energy transfer and charge transfer quenching.

The first mechanism—the energy transfer quenching—is the reverse reaction by which singlet oxygen is formed. The formation of triplet oxygen (^3^O_2_) and triplet quencher (^3^Q) occurs from the singlet states of oxygen and quencher (Equation 4):

(4) O 12+Q 1→O 32+Q 3

On the other hand, singlet oxygen is an important intermediate species in oxidation processes that are detrimental to biomolecules. It is capable of reacting with a wide range of biological molecules such as DNA, proteins, and lipids (Kasha and Khan, 1970). Furthermore, ^1^O_2_ is known as one of the reactive oxygen species that can be generated in the respiratory chain in aerobic organisms. The toxic nature of this species has led to a large number of studies focused on counteracting its effects through different strategies such as physical scavengers that act according to the energy transfer mechanism. In fact, carotenoids are known as highly effective quenchers even at very low concentrations (Foote and Denny, 1968). According to these studies, a single β-carotene molecule can deactivate between 250 and 1,000 singlet oxygen molecules. This deactivation could only be effective if the triplet state of β-carotene has an energy level below 22 kcal. This parameter seems to be accessible for the long-chain polyenes which have between 9 and 11 double bonds in conjugation. If the chain length consists of 7 or fewer double bonds, the molecules will act as inefficient quenchers (Kearns, 1971). The quenching of singlet oxygen by carotenoids depends on the energy difference between the lowest unoccupied (LUMO) and the highest occupied molecular orbital (HOMO), and on the ionization energy. Although this mechanism is efficient in deactivating ^1^O_2_, it is not the most appropriate for electrochemical systems (Petit et al., 2019).

The second mechanism consists in the interaction between the electron-deficient ^1^O_2_ molecule and electron donors to form a charge transfer complex. After the relaxation, dissociation of the adduct—with the recombination of the charges—generally leads to unchanged quencher and triplet oxygen (ground-state dioxygen). These types of inhibitors are amines, phenols, some natural complexes, sulfides, iodide, and it is the case of the azide anion. The azide radical drives the formation of the charge-transfer complex $O_{2}^{-}\cdots N_{3}$ through the following reaction (Harbour and Issler, 1982):

(5) O 12+N3−→{O2−⋯N3·}→O2−+N3·

Azide could be an interesting quencher for these energy storage systems, as recently demonstrated for both Li and Na-O_2_ batteries (Córdoba et al., 2019; Lozano et al., 2020). However, due to the explosive potential and safety concerns, azide should be avoided whenever possible if there is a reasonable alternative.

As mentioned, amines can also act as electron-rich physical quenchers by interacting singlet oxygen with the lone electron pair of the amine. Specifically, the activity of the PeDTFSI (1-pentyl-1,4-diazabicyclo[2.2.2]octan-1-ium bis(trifluoromethane)sulfon-imide) salt as quencher has been demonstrated by partial charge transfer (Petit et al., 2019). This ionic liquid, miscible with glymes (usual solvent in MaBs) and stable even at potentials of 4.2 V (vs. Li^+^/Li), is capable of reducing by 14% the amount of side products through efficient quenching of ^1^O_2_. Another system which was recently studied is based on the use of TEMPOImILs (2,2,6,6-tetramethylpiperidine-1-oxyl (TEMPO)-substituted imidazolium ionic liquids) as solvent and redox mediator (Tkacheva et al., 2020). In this work, the presence of nitroxy radicals facilitated the ^1^O_2_ suppression activity and hence 50 cycles were achieved in a Li-O_2_ battery; however, the quencher action of this ionic liquid has not yet been demonstrated.

It is challenging to establish the specific characteristics that an effective quencher must present, since its activity is highly dependent on other parameters such as the solvent that will affect the kinetics of the reaction. In any case, there are a series of common features that a molecule must present:

- Compatibility with all the battery components, ensuring that it does not react chemically or electrochemically with the electrodes, the electrolyte or the current collectors.
- Stability in the battery environment and inert toward the reaction products (M_x_O_2_), without interfering in the fundamental processes of the MaB.
- Miscibility with the aprotic solvent in order to avoid a possible agglomeration of the quencher that could affect singlet oxygen deactivation kinetics. Additionally, the quencher must be inactive toward the selected sodium or lithium salt.
- In the charge transfer complex formation mechanism, it is important to control the oxidation potential of the quencher. This potential can be tuned through the correct selection of the molecular structure and the substituents (nature and position in the molecule).

The molecular structure of the quencher plays an important role, since it affects the kinetics of the deactivation process and can influence the oxidation potential. Despite the fact that there are different studies on the effect of the structure on different families of molecules, to date, a criterion that can be applied to all systems has not been established.

There is still a long path for the design of an ideal quencher to avoid the devastating effects of singlet oxygen in metal-O_2_ batteries. Although there are already some works that analyze the role of certain molecules as singlet oxygen quenchers, this research is still in the early stages of its development. It is necessary to carry out an in-depth study that helps establish clear criteria on the characteristics of these molecules in addition to delving into the action mechanisms. Finally, it is of fundamental importance to clarify the real role of quenchers on battery performance, offering clear evidence of improved cyclability due to decreased parasitic reactions involving singlet oxygen.

## Conclusions

Metal-O_2_ batteries are considered a highly promising technology for the next generation of advanced batteries. However, there are a number of challenges that must be overcome before widespread commercialization is possible such as irreversible parasitic reactions. These reactions have been shown to be intimately linked to the presence of the singlet oxygen reactive species—generated by disproportionation of the superoxide anion. It is, therefore, essential to design novel quenchers that are electrochemically harmless to the battery and capable of interacting with singlet oxygen without consuming oxygen in the process or producing intermediate species that interact with the environment of the battery. Based on this, the most promising strategy is based on the development of physical quenchers that act through charge transfer mechanisms. Over the last few months, the activity of a small number of molecules (azide and special ionic liquids) as singlet oxygen quenchers in MaBs has been explored, obtaining highly promising results. However, there is still a long path to develop the ideal system that can avoid the devastating effects of this highly reactive molecule.

## Author Contributions

All authors listed have made a substantial, direct and intellectual contribution to the work, and approved it for publication.

## Conflict of Interest

The authors declare that the research was conducted in the absence of any commercial or financial relationships that could be construed as a potential conflict of interest.
